# Gastrointestinal and Extra-Intestinal Manifestations of Childhood Shigellosis in a Region Where All Four Species of *Shigella* Are Endemic

**DOI:** 10.1371/journal.pone.0064097

**Published:** 2013-05-17

**Authors:** Wasif A. Khan, Jeffrey K. Griffiths, Michael L. Bennish

**Affiliations:** 1 Centre for Vaccine Sciences, International Centre for Diarrhoeal Disease Research, Bangladesh (icddr, b), Dhaka, Bangladesh; 2 Department of Public Health and Community Medicine, School of Medicine, Tufts University School of Medicine, Boston, Massachusetts, United States of America; 3 Mpilonhle, Mtubatuba, South Africa; UCL Institute of Child Health, University College London, United Kingdom

## Abstract

**Objective:**

To determine the clinical manifestations and outcome of shigellosis among children infected with different species of *Shigella*.

**Methods:**

We identified all patients <15 years infected with *Shigella* admitted to the icddr, b Dhaka hospital during one year. Study staff reviewed admission records and repeated the physical examinations and history of patients daily.

**Results:**

Of 792 children with shigellosis 63% were infected with *S. flexneri*, 20% with *S. dysenteriae* type 1, 10% with *S. boydii*, 4% with *S. sonnei*, and 3% with *S. dysenteriae* types 2–10. Children infected with *S. dysenteriae* type 1, when compared to children infected with other species, were significantly (P<0.05) more likely to have severe gastrointestinal manifestations: grossly bloody stools (78% vs. 33%), more stools in the 24 h before admission (median 25 vs. 11), and rectal prolapse (52% vs. 15%) - and extra-intestinal manifestations - leukemoid reaction (22% vs. 2%), hemolytic-uremic syndrome (8% vs. 1%), severe hyponatremia (58% vs. 26%) and neurologic abnormalities (24% vs. 16%). The overall fatality rate was 10% and did not differ significantly by species. In a multiple regression analysis young age, malnutrition, hyponatremia, lesser stool frequency, documented seizure, and unconsciousness were predictive of death.

**Conclusions:**

Both severe intestinal disease and extra-intestinal manifestations of shigellosis occur with infection by any of the four species of *Shigella*, but are most common with *S. dysenteriae* type 1. Among these inpatient children, the risk of death was high with infection of any of the four Shigella species.

## Introduction

Shigellosis remains a major cause of morbidity and mortality among children in developing countries, and is also an important cause of morbidity in industrialized countries. [1], [2], [3], [4], [5] Clinical descriptions of bacillary dysentery were published soon after the identification by Shiga in 1897 of the organism now known as *Shigella dysenteriae* type 1, [6] and subsequent identifications of other species of *Shigella* by Flexner, Sonne, and Boyd [7].

Most clinical descriptions of shigellosis have focused on a single complication, or on the one or two serotypes that are prevalent in a single clinical setting, thus making direct comparisons of clinical manifestations of infections caused by different serotypes difficult. [8], [9] Few regions have endemic infection with all four species of *Shigella* simultaneously, or the ability to identify and study the problem if they do.

Bangladesh is an exception. Although severe dysentery and extra-intestinal manifestations – including hemolytic-uremic syndrome (HUS), [10] leukocytosis [11] and intestinal obstruction [12] – are thought to be more frequent with *S. dysenteriae* type 1 infection as a consequence of its toxin production, [13] the relative frequency of other complications - such as convulsions, [14] hypoglycemia, [15] and sepsis [16] - is either unknown or thought to be more frequent in species of Shigella other than *S. dysenteriae* type 1. In this report we compare clinical manifestations – both intestinal and extra-intestinal – and outcome in 792 children admitted with *Shigella* to an urban diarrhea disease treatment centre in Bangladesh.

## Methods

### Ethics Statement

This study was approved by the Ethical Review Committee (ERC) of the icddr, b, which waived the need for signed informed consent as all information was obtained from the standard medical record or as part of standard medical care. Information was recorded on a case report form that was expunged of any identifiers that would have linked the information to an individual patient.

### Patient Recruitment

The study was conducted at the Dhaka hospital of the icddr, b in Dhaka, Bangladesh, which provides care to patients with diarrhea. Most of the patients are treated as outpatients, or in a short stay (<24 hours) ward, where the focus is on hydration. Approximately 6% of patients who have more complicated disease are admitted to an inpatient ward where more intensive diagnostic and therapeutic care is available. Stool or rectal swab samples for identification of common enteric bacterial pathogens, including *Shigella,* are obtained from a systematic 2% sample of outpatients (during this study and until 1995 it was 4% sampling), and on all patients admitted to the inpatient unit.

Patients described in this study were enrolled in the 12-months from March 1987 through February 1988. Patients infected with *Shigella* were identified by daily review of the microbiology laboratory records of admission stool or rectal swab samples.

### Information Obtained

A systematic set of information was obtained from all patients who had *Shigella* isolated from a stool or rectal swab sample. Using a standard case report form study physicians obtained demographic and historical information, and physical examination findings, by reviewing the charts of patients infected with *Shigella* and by interviewing patients, or their parents or guardians, to confirm or complete the history of illness obtained by the admitting physician. Study physicians also recorded the results of all laboratory tests that were done, interval histories, and the results of repeat physical examinations. All patients who were still in the hospital at the time *Shigella* was isolated had physical examinations repeated by study physicians, and daily interval histories in addition to those done by the ward physician. Clinical management was under the direction of the attending physician.

Patient outcome was classified as discharged improved, discharged against medical advice, transferred to another health facility, or died in the hospital. Nutritional status was determined using United States National Center for Health Statistics growth charts. [17] All laboratory tests were performed using standard methods previously described [18].

### Statistical Analysis

Data were entered onto a computer database using StatPac Gold Version 3.2 (Walonick Associates, Minneapolis, Minnesota). Data analysis was performed using the Statistical Package for Social Sciences, versions 12.0 for Windows, (SPSS, Chicago, IL), and EpiInfo 2003 version 3.3.2 (Centers for Disease Control and Prevention, Atlanta, Georgia, USA). In analyzing differences by species and serotype, we compared *S. dysenteriae* type 1 infected patients with all other Shigella-infected patients, and also compared the groups individually. The significance of differences in proportions was tested by the chi-square test with continuity correction, or Fisher’s exact test if an expected cell size was <5. The significance of differences between continuous variables in two groups was tested with Students *t* test if the data were normally distributed, or the Mann-Whitney U test if the data were not normally distributed. For normally distributed continuous variables involving three or more groups an analysis of variance (ANOVA) was first used to test the significance of differences. Differences between individual groups were then tested for significance using Scheffe’s procedure if the overall F statistic was ≤0.20. For non-normal continuous variables involving three or more groups the Kruskal-Wallis test was used, and the Mann-Whitney U test was used to compare differences between two groups.

Factors independently predictive of death were determined using a multiple logistic regression analysis. Variables that in bivariate analysis had a P<0.10 when tested for association with death, or that could biologically be plausibly associated with death (infected with *Shigella dysenteriae* type 1 and stool frequency before admission) were entered into the regression equation and eliminated in a backward stepwise fashion if the probability associated with the likelihood ratio statistic exceeded 0.05. Blood glucose and serum protein results, though associated with death in bivariate analyses, were excluded from the multiple logistic regression analysis because these tests were performed on only a limited number of patients. Multivariate odds ratios and confidence intervals in the final logistic regression equation were calculated from the coefficient of the multiple regression models.

## Results

### Patient Population

During the one-year study *Shigella* was identified in a stool or rectal swab sample of 863 (14%) of the 6,290 patients admitted to the inpatient unit. This analysis focuses on the 792 (92%) of the 863 inpatients with *Shigella* infection who were children less than 15 years.

To determine the proportion of children with *Shigella* infection presenting as outpatients who were admitted to the inpatient unit, we extrapolated from data from the systematic surveillance system. 83,402 patients came to the icddr, b Dhaka hospital for care during the study period, of whom 55,644 (67%) were <15 years. *Shigella* was isolated from a stool or rectal swab sample of 209 (9.1%) of the 2,292 patients <15 years of age entered in the 4% systematic sample of outpatients. If this proportion is extrapolated to all outpatients <15 years of age, an estimated 5,074 outpatients were children <15 years of age infected with *Shigella*. Seven-hundred ninety-two inpatients with *Shigella* infection <15 years made for an admission rate of 16% for outpatients infected with *Shigella*, a rate of admission significantly (P<0.001) higher than the 10% rate for children not infected with *Shigella*. Shigellosis accounted for 14% (792/5,745) of patients <15 years of age admitted to the inpatient unit.

### Species of *Shigella* Isolated from Inpatients

*S. flexneri* was the most common species isolated, accounting for 504 (64%) of the 792 *Shigella* infections in inpatients <15 years. *S. dysenteriae* type-1 was isolated from 157 (20%) of the patients with culture-confirmed shigellosis (Table 1).

**Table 1:** Isolation of *Shigella* Species by Age Group of Patients Admitted to the Dhaka Hospital of the ICDDR, B.

Age group	*S. dysenteriae* type 1 (n = 165)	*S. dysenteriae* type 2–10(n = 28)	*S. flexneri* (n = 555)	*S. boydii* (n = 85)	*S. sonnei* (n = 30)	P for overall comparison
**<1 year**	31 (19)	6 (21)	217 (39)	34 (40)	18 (60)	<0.001*
**1 to 5 years**	108 (65)	15 (54)	259 (47)	38 (45)	11 (37)	<0.001†
**>5 to <15 years**	18 (11)	3 (11)	28 (5)	5 (6)	1 (3)	0.069
**≥15 years**	8 (5)	4 (14)	51 (9)	8 (9)	0	0.117

Values are n (% of patients in age group).

* P<0.040; S. dysenteriae type 1 versus S. flexneri, S. boydii or S.sonnei; S. dysenteriae types 2–10 versus S. sonnei; S. flexneri versus S. sonnei.

† P<0.006: S. dysenteriae type 1 versus S. flexneri, S. boydii or S. sonnei.

Three-hundred six (39%) of the children <15 years of age with shigellosis were below 1 year of age (including 5 neonates), 431 (54%) were 1–5 years, and 55 (6.9%) were >5 years. *S. dysenteriae* type 1 infection was significantly (P<0.001) less common, and *S. flexneri* significantly more common (P<0.001), in infants than in older children (Table 1). Median age for those infected with *S. dysenteriae* type 1 was 24 months versus 13 months for those infected with *S. flexneri* or *S. boydii* and 8 months for those infected with *S. sonnei* (P<0.020 for all comparisons between *S. dysenteriae* type 1 and each of the three other species). Patients infected with *S. dysenteriae* types 2–10 (median age 26 months) were significantly older (P = 0.018), and those infected with *S. sonnei* were significantly younger (P = 0.002) than those infected with *S. flexneri*.

### Admission Demographic and Clinical Characteristics

Admission demographic and clinical features that differed significantly (P<0.05) by infecting species in addition to age were body temperature and weight-for-age (Table 2). Children infected with *S. dysenteriae* type 1 were significantly more likely to be febrile than those infected with *S. flexneri*, and were significantly better nourished.

**Table 2:** Admission Clinical Characteristics of 792 Inpatients <15 Years by Species of *Shigella.*

Characteristic	*S. dysenteriae* type 1 (n = 157)	*S. dysenteriae*type 2–10 (n = 24)	*S. flexneri*(n = 504)	S. *boydii*(n = 77)	*S. sonnei*(n = 30)	P for overallcomparison	All patients withnon-*S. dysenteriae*type 1 infection(n = 635)	P for *S. dysenteriae* type 1 versus non-*S. dysenteriae* type 1 infection
**Age, m (median, 25^th^, 75^th^ quartiles)**	24 (13, 43)	26 (10, 50)	13 (7, 30)	13 (6, 30)	8 (4, 15)	<0.001*	13 (6, 30)	<0.001
**Male**	92 (59)	15 (63)	285 (57)	43 (56)	18 (60)	0.958	361 (57)	0.719
**Duration of illness, d (median, 25^th^,** **75^th^ centiles)** †	6 (4, 9)	2 (1, 10)	5 (3, 14)	5 (3, 15)	5 (3, 10)	0.051‡	5 (3, 14)	0.405
**Severe dehydration**	4 (3)	0	23 (5)	3 (4)	3 (10)	0.304	29 (5)	0.362
**Temperature ≥ 38.0°C**	107 (68)	12 (50)	265 (53)	27 (35)	15 (50)	<0.001§	319 (50)	<0.001
**Weight for age, % of median** **(mean ± SD)** || ¶	62±14	65±7	57±15	55±13	59±16	<0.001**	57±14	<0.001
**Pedal edema**	27 (17)	4 (17)	88 (18)	11 (14)	2 (7)	0.603	105 (17)	0.842

Values are n (%) unless noted.

* P<0.020: *S. dysenteriae* type 1 versus *S. flexneri*, *S. boydii*, or *S. sonnei*; *S. dysenteriae* types 2–10 versus *S. flexneri*, *S. boydii* or *S. sonnei*; *S. flexneri* versus *S. sonnei*. P<0.008: *S. dysenteriae* type 1 versus non-*S. dysenteriae* type 1.

† Duration of illness data were missing for 2/157 patients in the *S. dysenteriae* type 1 group, 5/504 patients in the *S. flexneri* group, and 1/77 patient in the *S. boydii* group.

‡ P<0.009: *S. dysenteriae* types 2–10 versus *S. dysenteriae* type 1, *S. flexneri*, *S. boydii* or *S. sonnei.*

§ P<0.007: *S. dysenteriae* type 1 versus *S. flexneri or S. boydii*; *S. flexneri* versus *S. boydii.*

|| Weight-for-age was calculated as a percentage of the United States National Center for Health Statistics median weight-for-age^17.^

¶ Weight-for-age data were missing for 5/157 patients in the *S. dysenteriae* type 1 group, 1/24 patient in the *S. dysenteriae* type 2–10 group, and 11/504 patients in the *S. flexneri* group.

** P<0.002: *S. dysenteriae* type 1 versus *S. flexneri or S. boydii*; *S. dysenteriae* types 2–10 versus *S. flexneri* or *S. boydii.*

### Intestinal Manifestations of Infection

Children with *S. dysenteriae* type 1 infection had a more severe colitis than children infected with other species or serotypes of *Shigella*. Those infected with *S. dysenteriae* type 1 more commonly had grossly bloody stools (78% versus 33%), more erythrocytes and leukocytes on microscopic examination of stool, more often had rectal prolapse (52% versus 15%) or abdominal tenderness (26% versus 6%), and a higher number of stools in the 24 hours before admission to hospital (median 25 versus 11) (P<0.001 for all comparisons with other species) (Table 3). Severe dehydration was rare in these children, occurring in only 33 (4%) of Shigella-infected patients. Of those who were severely dehydrated, the median creatinine concentration was 118 mmol/l, compared to 87 mmol/l in children without severe dehydration.

**Table 3:** Intestinal Manifestation of Shigellosis in 792 Inpatients <15 Years by Species of *Shigella*.

Clinical characteristic	*S. dysenteriae*type 1 (n = 157)	*S. dysenteriae.* type 2–10 (n = 24)	*S. flexneri*(n = 504)	*S. boydii*(n = 77)	*S. sonnei*(n = 30)	P – overall comparison	All patients with non-*S. dysenteriae* type 1 infection (n = 635)	P for *S. dysenteriae* type 1 versus non-*S. dysenteriae* type 1 infection
**Grossly bloody stool** *	122 (78)	5 (21)	180 (36)	19 (25)	6 (20)	<0.001†	210 (33)	<0.001
**Number of stools in 24 h before admission, (median, 25^th^, 75^th^ centiles)** ‡	25 (12, >100)	10 (6, 15)	12 (7, 20)	10 (6, 15)	10 (7, 15)	<0.001§	11 (7, 20)	<0.001
**Rectal prolapse** ||	79 (52)	2 (8)	86 (18)	4 (5)	1 (3)	<0.001¶	93 (15)	<0.001
**Abdominal examination**								
** Decreased or absent bowel sounds**	4 (3)	0	5 (1)	3 (4)	0	0.215	8 (1)	0.268
** Abdominal distention**	21 (13)	2 (8)	57 (11)	18 (23)	2 (7)	0.036**	79 (12)	0.856
** Abdominal tenderness**	41 (26)	3 (13)	27 (5)	5 (7)	1 (3)	<0.001††	36 (6)	<0.001
**Stool microscopic examination**								
** Leukocyte count** ‡‡								
** ≤10 per high-powered field**	10 (7)	1 (4)	64 (14)	25 (38)	3 (12)	<0.001§§	93 (16)	<0.001
** 11–50 per high-powered field**	35 (24)	10 (44)	151 (33)	21 (31)	15 (63)		197 (35)	
** >50 per high-powered field**	101 (69)	12 (52)	241 (53)	21 (31)	6 (25)		280 (49)	
** Erythrocyte count** §§								
** 0 per high-powered field**	11 (7)	8 (35)	97 (21)	32 (48)	4 (17)	<0.001|| ||	141 (25)	<0.001
** 1–10 per high-powered field**	33 (23)	7 (30)	188 (41)	24 (36)	17 (71)		236 (41)	
** 11–50 per high-powered field**	38 (26)	6 (26)	113 (25)	7 (10)	2 (8)		128 (22)	
** >50 per high-powered field**	64 (44)	2 (9)	60 (13)	4 (6)	1 (4)		67 (12)	

Values are n (%), unless noted.

* Stool character data were missing for 1/157 patient in the *S. dysenteriae* group, 1/504 in the *S. flexneri* group, and 1/77 in the *S. boydii* group.

† P<0.001:*S. dysenteriae* type 1 versus *S. dysenteriae* type 2–10, *S. flexneri*, *S. boydii* or *S. sonnei.*

‡ Stool frequency data were missing for 3/157 patients in the *S. dysenteriae* type 1 group, 9/504 patients in the *S. flexneri* group, 5/77 patients in the *S. boydii* group, and 1/30 patient in the *S. sonnei* group.

§ P<0.001: *S. dysenteriae* type 1 versus *S. dysenteriae* type 2–10, *S. flexneri*, *S. boydii*, or *S. sonnei.*

|| Rectal prolapse data were missing for 6/157 patients in the *S. dysenteriae* type 1 group, 13/504 patients in the *S. flexneri* group, 3/77 patients in the *S. boydii group*, and 1/30 patient in the *S. sonnei* group.

¶ P<0.040: *S. dysenteriae* type 1 versus *S. dysenteriae* type 2–10, *S. flexneri*, *S. boydii*, or *S. sonnei*; *S. flexneri* versus *S. boydii.*

** P = 0.006, *S. flexneri* versus *S. boydii.*

†† P<0.015: *S. dysentereriae* type 1 versus *S. flexneri, S. boydii*, or *S. sonnei.*

‡‡ Stool leukocyte count data were missing for 11/157 patients in the *S. dysenteriae* type 1 group, 1/24 patient in the *S. dysenteriae* type 2–10 group, 48/504 patients in the *S. flexneri* group, 10/77 patients in the *S. boydii* group, and 6/30 patients in the *S. sonnei* group.

§§ P<0.020: *S. dysenteriae* type 1 versus *S. flexneri*, *S. boydii*, or *S. sonne*; *S. dysenteriae* type 2–10 versus *S. boydii*; *S. flexneri* versus *S. boydii*, or *S. sonnei*; *S. boydii* versus *S.sonnei.*

§§ Stool erythrocyte count data were missing for 11/157 patients in the *S. dysenteriae* type 1 group, 1/24 patient in the *S. dysenteriae* type 2–10 group, 46/504 patients in the *S. flexneri* group, 10/77 patients in the *S. boydii* group, and 6/30 patients in the *S. sonnei* group.

|| || P<0.050: *S. dysenteriae* type 1 versus *S. dysenteriae* type 2–10, *S. flexneri*, *S. boydii*, or *S. sonnei*; *S. dysenteriae* type 2–10 versus *S. sonnei*; *S. flexneri* versus *S. boydii*, or *S. sonnei*; *S. boydii* versus *S.sonnei.*

### Extra-intestinal Manifestation

On admission, or during their hospital stay, children infected with *S. dysenteriae* type 1, in comparison with children infected with other species or serotypes of *Shigella*, were significantly (P<0.035) more likely to have leukemoid reaction (22% versus 2%), HUS (8% versus 1%), hyponatremia as manifested by a serum sodium <126 mmol/l (58% versus 26%), hypoproteinemia <60 gm/l (81% versus 62%), and any neurological abnormality (24% versus 16%). Features that approached statistical significance were convulsions during hospitalization (8% versus 4%, P = 0.079); and unconsciousness (13% versus 8%, P = 0.121) (Table 4).

**Table 4:** Extra-intestinal Manifestations of Shigellosis in 792 Inpatients <15 Years by Species of *Shigella.*

Characteristics	*S. dysenteriae*type 1 (n = 157)	*S. dysenteriae* type2–10 (n = 24)	*S. flexneri*(n = 504)	*S. boydii*(n = 77)	*S. sonnei*(n = 30)	P – overallcomparison	All patients with non-*S. dysenteriae* type 1infection (n = 635)	P for *S. dysenteriae* type 1 versus non-S. dysenteriae type 1 infection
**Altered neurological state**								
** Convulsion**								
** Documented in hospital**	13 (8)	2 (8)	26 (5)	0	0	0.052*	28 (4)	0.079
** By history (before admission)**	4 (3)	2 (8)	12 (4)	5 (7)	1 (3)	0.178	20 (3)	1.0
** Unconsciousness**	20 (13)	4 (17)	45 (9)	2 (3)	2 (7)	0.083	53 (8)	0.121
** Any neurologic abnormality**	37 (24)	8 (33)	83 (17)	7 (9)	3 (10)	0.009†	101 (16)	0.032
**Leukemoid reaction (peripheral blood white cells >40,000/mm^3^)** ‡	32 (22)	0	10 (2)	0	2 (7)	<0.001§	12 (2)	<0.001
**Hematocrit, (mean ± SD)** ||	36±7	37±5	34±6	34±6	35±7	0.001^¶^	34±6	0.001
**Creatinine (mmol/L; median & interquartile)** **	94 (78, 135)	107 (72, 160)	84 (67, 112)	88 (73, 116)	77 (68, 137)	0.024††	86 (68, 114)	0.003
**Hemolytic-uremic syndrome** ‡‡	13 (8)	0	4 (1)	2 (3)	2 (7)	<0.001§§	8 (1)	<0.001
**Severe hyponatremia (<126 mmol/L)** || ||	86 (58)	3 (13)	129 (27)	14 (21)	5 (18)	<0.001¶¶	151 (26)	<0.001
**Hypoglycemia (<2.2 mmol/L)** ***	7 (19)	1 (20)	21 (21)	5 (36)	0	0.521	27 (21)	0.797
**Hypoproteinemia (<60 gm/L)** †††	42 (81)	4 (44)	82 (65)	12 (48)	5 (71)	0.030‡‡‡	103 (62)	0.011
**Bacteremia** §§§	15 (13)	2 (12)	47 (14)	5 (11)	5 (25)	0.647	59 (14)	0.858

Values are worst (most abnormal) during hospital stay. Values are n (%).

* P<0.040: *S. dysenteriae type 1* versus *S. boydii*, *S. flexneri* versus *S. boydii.*

† P<0.05: *S. dysenteriae* type 1 versus *S. boydii; S. dysenteriae* types 2–10 versus *S. flexneri*, *S. boydii* or *S. sonnei.*

‡ Blood leukocytes data were missing for 10/157 patients in the *S. dysenteriae* type 1 group, 26/504 patients in the *S. flexneri* group, 6/77 patients in the *S. boydii* group, and 1/30 patient in the *S. sonnei* group.

§ .P<0.001: *S. dysenteriae* type 1 versus *S. dysenteriae* types 2–10, *S. flexneri, S. boydii.*

|| Hematocrit data were missing for 4/157 patients in the *S. dysenteriae* type 1 group, 23/504 patients in the *S. flexneri* group, and 5/77 patients in the *S. boydii* group.

¶ ¶ ¶ P<0.019: *S. dysenteriae* type 1 versus *S. flexneri* or *S. boydii*; *S dysenteriae* type 2–10 versus *S. flexneri* or *S. boydii.*

** Serum Creatinine data were missing for 53/157 patients in the *S. dysenteriae* type 1 group, 9/24 patients in the *S. dysenteriae* types 2–10 group, 304/504 patients in the *S. flexneri* group, 51/77 patients in the *S. boydii* group, and 20/30 patients in the *S. sonnei.*

†† P = 0.001: *S. dysenteriae* type 1 versus *S. flexneri.*

‡‡ Diagnosis of hemolytic-uremic syndrome required three criteria to be met: 1) an absolute packed cell volume <20%; or 2) a decrease in absolute packed cell volume of >10% in 24 hours; or 3) ≥0.5% schistocytes on a peripheral blood; and 4) serum creratinine, >180 mmol/L.

§§ P<0.050: *S. dysenterie* type 1 vesus *S. flexneri*; *S. flexneri* versus *S. sonnei.*

|| || Serum sodium data were missing for 9/157 patients in the *S. dysenteriae* type1 group, 34/504 patients in the *S. flexneri* group, 9/77 patients in the *S. boydii* group, and 2/30 patients in the *S. sonnei.*

¶¶ P<0.001: *S. dysenteriae* type 1 versus *S. dysenteriae* type 2–10, *S. flexneri*, *S. boydii* or *S. sonnei.*

*** Blood glucose data were missing for 121/157 patients in the *S. dysenteriae* type 1 group, 19/24 patients in the *S. dysenteriae* types 2–10 group, 402/504 patients in the *S. flexneri* group, 63/77 patients in the *S. boydii* group, and 25/30 patients in the *S. sonnei* group.

††† Serum protein data were missing for 105/157 patients in the *S. dysenteriae* type 1 group, 15/24 patients in the *S. dysenteriae* types 2–10 group, 378/504 patients in the *S. flexneri* group, 52/77 patients in the *S. boydii* group; and 23/30 patients in the *S. sonnei* group.

‡‡‡ P<0.035: *S. dysenteriae* type 1 versus *S. dysenteriae* types 2–10, or *S. boydii.*

§§§ Blood culture data were missing for 42/157 patients in the *S. dysenteriae* type 1 group, 5/24 patients in the *S. dysenteriae* types 2–10 group, 172/504 patients in the *S. flexneri* group, 32/77 patients in the *S. boydii* group; and 10/30 patients in the *S. sonnei* group.

### Outcome

Mortality in patients with *Shigella* infection was high - 83 (10%) of the 792 patients less than 15 years died (Table 5). Mortality was high for all species and serotypes, except for those infected with *S. dysenteriae* type 2–10, only 1 (4%) of whom died. There were no deaths in the 71 patients 15 years or older infected with *Shigella*.

**Table 5:** Outcome of 792 patients <15 Years by Species of *Shigella*.

Age group	*S. dysenteriae* type 1 (n = 157)	*S. dysenteriae* types 2–10 (n = 24)	*S. flexneri* (n = 504)	*S. boydii* (n = 77)	*S. sonnei* (n = 30)	P, overall comparison	All patients with non-*S. dysenteriae* type 1 infection (n = 635)	P for *S. dysenteriae* type 1 versus non-*S. dysenteriae* type 1 infection
**Discharged improved**	97 (62)	19 (79)	331 (65)	59 (77)	19 (63)	0.131	428 (67)	0.215
**Discharged against medical advice**	32 (20)	4 (17)	91 (18)	8 (10)	7 (23)	0.374	110 (17)	0.436
**Referred**	11 (7)	0	29 (6)	2 (3)	0	0.262	31 (5)	0.387
**Died in hospital**	17 (11)	1 (4)	53 (11)	8 (10)	4 (13)	0.861	66 (10)	0.989

Values are n (% of patients by species).

### Factors Associated with Mortality in a Multiple Logistic Regression Analysis

Variables included in the regression analysis were: age; stool frequency and duration of illness before admission; admission dehydration status and abdominal distention; weight for age; leukemoid reaction; infection with *S. dysenteriae* type 1 serotype; hyponatremia; documented seizure and unconsciousness. Factors significantly (P<0.05) associated with death were younger age, lower stool frequency before admission, poor nutrition, hyponatremia, documented seizure and unconsciousness (Table 6).

**Table 6:** Features Predictive of Death in 792 Patients <15 Years with Shigellosis in a Multiple Logistic Regression Analysis.

Characteristic	Odds Ratio	95% Confidence Interval	P
**Age, m**	0.966	0.947–0.986	**0.001**
**Number of stools before admission**	0.976	0.961–0.991	**0.002**
**Percent of median weight-for-age** *	0.957	0.932–0.983	**0.001**
**Serum sodium <126 mml/L**	3.75	1.93–7.29	**<0.001**
**Convulsion**	14.60	5.31–40.13	**<0.001**
**Unconsciousness**	45.40	17.48–117.96	**<0.001**

* Median weight-for-age is based on United States National Center for Health Statistics standard^17.^

Analysis was limited to 451 (86%) of the 525 patients discharged improved, and 65 (78%) of the 83 patients who died for whom information on each of the variables entered into the final iteration of the multiple logistic regression analysis was available.

Variables significant (P<0.05) in the bivariate analysis but excluded from the multiple logistic regression analysis because information was available for a limited number of the 516 patients were blood glucose, available for 127 (25%) patients, and serum protein, available in 163 (32%) patients.

## Discussion

This is the largest study to date comparing clinical manifestations of shigellosis in all four species of *Shigella*. The results of this study emphasize both the distinctiveness of infections with *S. dysenteriae* type 1, and the ability of all species of *Shigella* to cause intestinal and extra-intestinal manifestations, and death, in a susceptible population.

In the 792 children in this study, children with *S. dysenteriae* type 1 infection were older than those with the most common infection, *S. flexneri*. This appears to not simply result from a selection bias of who is admitted to the inpatient unit, as during the study period outpatients with *S. dysenteriae* type 1 infection (median age 24 months) were also older than outpatients with S. *flexneri* (median age13 months). This older age of patients with *S. dysenteriae* type 1 infection is consistent with previous findings from community-based studies in Bangladesh and our previous study of infant shigellosis. [19], [20] It is not clear why *S. dysenteriae* type 1 affects older children when compared to *S. flexneri*. The age difference might result from differing patterns of transmission, but both are known to be transmissible with a very low inoculum. It may be that infection with one serotype of S. *flexneri* confers subsequent immunity against infection with other serotypes of *S. flexneri*, but provides lesser or no immunity to infection with other *Shigella* species. Most evidence suggests, however, that immunity is serotype, and not species, specific. [21] With multiple serotypes of *S. flexneri* circulating, the probability of exposure to *S. flexneri* in the first year of life might simply be greater than for *S. dysenteriae* type 1. Exposure to *S. dysenteriae* type 1 may also be less sustained because it occurs in periodic epidemics, and cohorts of children may not be exposed every year.

*S. dysenteriae* type 1 is known to more often cause clinical dysentery than infections with other species of *Shigella.* [19], [22] The results of this study make clear the clinical manifestations and magnitude of those differences. *S. dysenteriae* type 1 infected patients were more than twice as likely to have grossly bloody stools when compared to patients infected with other species of *Shigella*, and *S. dysenteriae* type 1 infection was more than three times as likely to cause rectal prolapse. Patients with *S. dysenteriae* type 1 infection had a stool frequency before admission more than twice that of other groups, and were more likely to have abdominal tenderness and fever. All of this is consistent with the role of Shiga toxin, which, among *Shigella*, is produced only by the *S. dysenteriae* type 1 serotype, in exacerbating mucosal destruction and in intensifying the inflammatory response, [13] including eliciting increased production of pro-inflammatory cytokines [23], [24].

Dehydration was not a common problem in these patients with shigellosis. Only 4% had severe dehydration diagnosed clinically, and serum creatinine concentrations were consistent with the clinical assessments. Thus despite the identification of three enterotoxins in *Shigella* - Shiga toxin (only found in *S. dysenteriae* type 1) [25] and Shigella enterotoxin 1 (ShET-1) and Shigella enterotoxin-2 (ShET-2) (both found predominantly in *S. flexneri* 2a) [26], [27] – that in animal models cause fluid secretion. The effect on fluid secretion of these toxins in infections in humans is clearly much less than for cholera toxin, or *Escherichia coli* heat stable or heat labile toxins, which much more commonly cause dehydration.

The hypoproteinemia was most severe in patients infected with *S. dysenteriae* type 1 and most likely results from the more severe colitis and protein loss in the gut that occur with *S. dysenteriae* type 1 infection. [28] The severity of the hypoproteinemia in the *S. dysenteriae* type 1 infected group is all the more remarkable given that by anthropometry they were better nourished than patients infected with other species of *Shigella*. This latter finding is at odds with a previous study of outpatients with shigellosis at the icddr, b [19] and may reflect a selection bias in types of patients admitted to the inpatient unit – with older, better nourished patients being more likely to be admitted if they have severe clinical dysentery.

Other extra-intestinal manifestations also more common in *S. dysenteriae* type 1 infection included alterations in consciousness, severe hyponatremia, leukemoid reaction, and HUS. Both leukemoid reaction and HUS are thought to be related to Shiga toxin production. In this study they occurred significantly more frequently in *S. dysenteriae* type 1 infection, though they were not restricted to *S. dysenteriae* type 1 infections. Thirteen patients with *S. dysenteriae* type 1 infection and eight patients with non-*S. dysenteriae* type 1 infections met the criteria for HUS. Although the etiologic trigger for HUS is presumed in most cases to be infection with Shiga toxin-producing organisms (either *S. dysenteriae* type 1, or Shiga toxin-producing *Escherichia coli*) there are childhood cases of HUS that are not associated with Shiga toxin-producing organisms. [29] Further studies are needed to understand more about the pathogenesis and clinical course of these patients with HUS caused by non-*S. dysenteriae* type 1 strains of *Shigella*.

Severe hyponatremia, though more often found with *S. dysenteriae* type 1 infection, was common in infections with all species of *Shigella*. This suggests that it is not solely a Shiga-toxin related phenomenon. We have previously shown that it is associated with altered consciousness in this group of patients. [14] The cause of hyponatremia during shigellosis is uncertain, but preliminary studies suggest that it may be due to inappropriate secretion of antidiuretic hormone [30].

During the one year of this study the death rate in patients with shigellosis was high –83 (10%) of the 792 children less than 15 years admitted to the inpatient unit died. Another 5% were transferred to another facility because of complications that could not be treated at the icddr, b Dhaka hospital, or left the hospital against medical advice. Although the outcome of these patients was not known, patients in both groups were often severely ill, and it is likely that a number of them also died. None of the 71 patients 15 years or older died, including 15 older than 60 years. Most of the factors predictive of death in this study – young age, poorer nutritional status, lower serum sodium, lower stool frequency, convulsions and unconsciousness - have been associated with death in shigellosis in previous studies (Table 7). [16], [20], [31], [32], [33], [34] The two studies examining risk factors for death that were not done in Bangladesh (one in Rwanda, one in Zimbabwe, and both restricted to *S. dysenteriae* type 1-infected patients) both found severe dehydration as predictive of death. [31], [32] In this and other studies dehydration was neither common nor associated with death. For reasons we cannot explain, stool frequency was inversely associated with risk of death.

**Table 7:** Summary of Previous Studies Examining Risk Factors for Death in Patients with Shigellosis.

Study Author, Reference	Country	Study Date	Age of Patients	Patients, n	Deaths, n (%)	*Shigella* Species	Predictors of Death	Odds ratio	Comment
**Bennish ** [**16**]	Bangladesh	1983	≤10 years	201	67 (34)	23% S. dysenteriae type 1,4% S. dysenteriae type 2–10,62% S. Flexneri, 6% S.boydii, 5% S. sonnei	Age in months	0.969	Case-control study conducted in hospitalized patients in an urban area
							Serum protein (g/l)	0.945	
							Altered consciousness	4.80	
							Thrombocytopenia	9.29	
**Mitra ** [**34**]	Bangladesh	1980	≤5 years	46	23 (50)	Not recorded	Female	4.30	Case-control study conducted in hospitalized patients in a rural area
							Signs of lower respiratory infection	24.0	
							Severe malnutrition (<60% of weight for age)	8.90	
**Huskins ** [**20**]	Bangladesh	1984–1988	≤3 months	121	26 (21)	9% *S. dysenteriae* type 1,3% *S. dysenteriae* type 2–10,59% *S. flexneri*, 21% *S. boydii*,8% *S. sonnei*	Gram-negative bacteremia	14.61	Prospective study of infants and young children in an urban hospital. Study patients were a subset of patients in the current study
							Ileus	93.83	
							Decreased bowel sounds	86.81	
							Serum sodium (mmol/L)	0.884	
							Serum protein concentration (g//L)	0.819	
							Number of erythrocytes on stool microscopic examination (high power field)	0.931	
**Nathoo ** [**32**]	Ziwakhanmbabwe	1993–1994	1 month to 12 years	312	95 (30)	100% *S. dysenteriae* type 1	Temperature (<36.0°C)	2.12	Study conducted in two urban tertiary referral hospitals. Data from 264 patients collected prospectively; data on 48 patients collectedRetrospectively
							Severe dehydration	1.70	
							Serum sodium (<120 mmol/L)	1.57	
							Serum potassium (>5.5 mmol/L)	1.41	
							Urea (>8 mmol/L)	1.74	
							Abdominal distention	1.67	
**Legros ** [**31**]	Rwanda	1994	All ages	849	108 (13)	100% *S. dysenteriae* type 1	Severe dehydration	2.79	Prospective study conducted in 10 rural hospitals
							Pedal edema	2.20	
							Age (<5 years or >50 years)	3.22	
							Use of nalidixic acid	8.66	
**Van den Broek ** [**33**]	Bangladesh	1993–1999	≤4 years	200	100 (50)	19% *S. dysenteriae* type 1, 81% *S. flexneri*	Altered consciousness	2.60	Case control study in urban hospital restricted to malnourished children (<60% of median weight-for- age)
							Pneumonia	2.50	
							Hypoglycemia (<3 mmol/L)	7.80	
							Temperature (<36.0°C)	5.70	
**Khan, this study**	Bangladesh	1987–1988	<15 years	792	83 (10)	20% *S. dysenteriae* type 1, 3% *S. dysenteriae* type 2–10, 63% *S. Flexneri*, 10% *S. boydii*, 4% *S. sonnei*	Age, m	0.966	Prospective study in an urban hospital
							Number. of stools before admission	0.976	
							Percent of median weight-for-age	0.957	
							Serum sodium, (<126 µmol/l)	3.75	
							Convulsion	14.60	
							Unconsciousness	45.40	

All studies used multivariate logistic regression analysis to determine factors predictive of death with the exception of Mitra (reference 34), which used bivariate analysis.

The high death rate seen in patients with shigellosis in developing countries such as Bangladesh contrasts dramatically with the rarity of deaths from shigellosis found in industrialized countries. [33], [35] Given that deaths occurred with all *Shigella* species, including those species – *S. sonnei* and *S. flexneri* - that cause infection in industrialized countries – the differential rates in mortality between rich and poor countries are most likely due to differences in host factors (including underlying nutritional status) and health care. [36] We have shown that early treatment (within 72 hours of the onset of illness) of *S. dysenteriae* type 1 virtually eliminates the risk of developing HUS. [37] In this study 68% of patients were ill for more than 72 hours before coming for care, and by that time many of the complications of illness - hypoproteinemia, alterations in consciousness, and HUS - were well established. Preventing the more serious intestinal and extra-intestinal manifestations of shigellosis, and death, requires both earlier treatment of clinical disease and improvements in nutritional conditions of children. Neither of these recommendations is easy to implement, as antimicrobial resistance in *Shigella* is increasingly common, [38] the health care systems required to provide early care continue to struggle in many developing countries, and the same conditions of poverty that lead to high rates of shigellosis also lead to the high rates of malnutrition seen in these poor communities [39].

Since Kiyoshi Shiga first identified the etiologic agent of bacillary dysentery in 1897 [40] there have been a number of descriptions of the clinical manifestations of illness caused by *Shigella*. [1], [9], [41], [42] Many of these reports have focused on *Shigella* infections and complications, usually rare, that occur at sites outside the gut, including the eye [41], [43] or the genitourinary tract. [41], [44], [45] We did not actively search for infections at these sites, nor did we have the longer term follow-up that would allow us to accurately ascertain the risk of post-infectious complications, such as Reiter’s syndrome, that have been infrequently reported to occur during shigellosis [46].

This study demonstrates that extra-intestinal manifestations of shigellosis are not confined to infection with *S. dysenteriae* type 1, as is commonly thought. The spectrum of severe intestinal and extra-intestinal complications that occur with all species of *Shigella* make this a challenging illness to treat effectively once complications have developed, especially in resource constrained settings and in malnourished hosts. Early treatment with an effective antimicrobial agent is likely to lessen both morbidity and mortality, especially the development of severe intestinal and extra-intestinal manifestations of illness. But increasing resistance to commonly used antimicrobial agents also makes this option an increasing challenge.

## References

[1] Bennish ML, Khan WA (2012) Shigellosis. In: Magill AJ, Ryan ET, Hill DR, Solomon T, editors. Hunter's Tropical Medicine and Emerging Infectious Diseases Ninth ed. Maryland: Elsevier Sanders. pp. 454–461.

[2] Ram PK, Crump JA, Gupta SK, Miller MA, Mintz ED (2008) Part II. Analysis of data gaps pertaining to Shigella infections in low and medium human development index countries, 1984–2005. Epidemiol Infect 136: 577–603. 17686195 10.1017/S0950268807009351 PMC2870860

[3] von Seidlein L, Kim DR, Ali M, Lee H, Wang X, et al (2006) A multicentre study of Shigella diarrhoea in six Asian countries: disease burden, clinical manifestations, and microbiology. PLoS Med 3: e353. 16968124 10.1371/journal.pmed.0030353 PMC1564174

[4] Kotloff KL, Winickoff JP, Ivanoff B, Clemens JD, Swerdlow DL, et al (1999) Global burden of Shigella infections: implications for vaccine development and implementation of control strategies. Bull World Health Organ 77: 651–666. 10516787 PMC2557719

[5] Niyogi SK (2005) Shigellosis. J Microbiol 43: 133–143. 15880088

[6] Shiga K (1898) Ueben den Dysenterie bacillus (*Bacillus dysenteriae*). Zentralbl Bakteriol Parasitenkd Abt I Org 24: 817–824.

[7] Hale TL (1991) Genetic basis of virulence in Shigella species. Microbiol Rev 55: 206–224. 1886518 10.1128/mr.55.2.206-224.1991 PMC372811

[8] Chopra M, Wilkinson D, Stirling S (1997) Epidemic shigella dysentery in children in northern KwaZulu-Natal. S Afr Med J 87: 48–51. 9063314

[9] Ashkenazi S (2004) Shigella infections in children: new insights. Semin Pediatr Infect Dis 15: 246–252. 15494948 10.1053/j.spid.2004.07.005

[10] Koster F, Levin J, Walker L, Tung KS, Gilman RH, et al (1978) Hemolytic-uremic syndrome after shigellosis. Relation to endotoxemia and circulating immune complexes. N Engl J Med 298: 927–933. 642973 10.1056/NEJM197804272981702

[11] Rahaman MM, JamiulAlam AK, Islam MR, Greenough WB 3rd (1975) Shiga bacillus dysentery associated with marked leukocytosis and erythrocyte fragmentation. Johns Hopkins Med J 136: 65–70. 1090770

[12] Bennish ML, Azad AK, Yousefzadeh D (1991) Intestinal obstruction during shigellosis: incidence, clinical features, risk factors, and outcome. Gastroenterology 101: 626–634. 1860627 10.1016/0016-5085(91)90518-p

[13] O'Loughlin EV, Robins-Browne RM (2001) Effect of Shiga toxin and Shiga-like toxins on eukaryotic cells. Microbes Infect 3: 493–507. 11377211 10.1016/s1286-4579(01)01405-8

[14] Khan WA, Dhar U, Salam MA, Griffiths JK, Rand W, et al (1999) Central nervous system manifestations of childhood shigellosis: prevalence, risk factors, and outcome. Pediatrics 103: E18. 9925864 10.1542/peds.103.2.e18

[15] Bennish ML (1991) Potentially lethal complications of shigellosis. Rev Infect Dis 13 Suppl 4S319–324. 2047657 10.1093/clinids/13.supplement_4.s319

[16] Bennish ML, Harris JR, Wojtyniak BJ, Struelens M (1990) Death in shigellosis: incidence and risk factors in hospitalized patients. J Infect Dis 161: 500–506. 2313128 10.1093/infdis/161.3.500

[17] CDC (2000) National Center for Health Statistics - Clinical Growth Charts. Available: http://www.cdc.gov/nchs/about/major/nhanes/growthcharts/clinical_charts.htm. Accessed 2005 Oct 15.

[18] Salam MA, Bennish ML (1988) Therapy for shigellosis. I. Randomized, double-blind trial of nalidixic acid in childhood shigellosis. J Pediatr 113: 901–907. 3054035 10.1016/s0022-3476(88)80029-5

[19] Ahmed F, Clemens JD, Rao MR, Ansaruzzaman M, Haque E (1997) Epidemiology of shigellosis among children exposed to cases of Shigella dysentery: a multivariate assessment. Am J Trop Med Hyg 56: 258–264. 9129527 10.4269/ajtmh.1997.56.258

[20] Huskins WC, Griffiths JK, Faruque AS, Bennish ML (1994) Shigellosis in neonates and young infants. J Pediatr 125: 14–22. 8021764 10.1016/s0022-3476(94)70115-6

[21] Jennison AV, Verma NK (2004) Shigella flexneri infection: pathogenesis and vaccine development. FEMS Microbiol Rev 28: 43–58. 14975529 10.1016/j.femsre.2003.07.002

[22] Faruque AS, Teka T, Fuchs GJ (1998) Shigellosis in children: a clinico-epidemiological comparison between Shigella dysenteriae type I and Shigella flexneri. Ann Trop Paediatr 18: 197–201. 9924557 10.1080/02724936.1998.11747947

[23] de Silva DG, Mendis LN, Sheron N, Alexander GJ, Candy DC, et al (1993) Concentrations of interleukin 6 and tumour necrosis factor in serum and stools of children with Shigella dysenteriae 1 infection. Gut 34: 194–198. 8432472 10.1136/gut.34.2.194 PMC1373969

[24] Thorpe CM, Smith WE, Hurley BP, Acheson DW (2001) Shiga toxins induce, superinduce, and stabilize a variety of C-X-C chemokine mRNAs in intestinal epithelial cells, resulting in increased chemokine expression. Infect Immun 69: 6140–6147. 11553553 10.1128/IAI.69.10.6140-6147.2001 PMC98744

[25] Keusch GT (1977) Bacterial toxins as virulence factors: Shiga bacillus dysentery viewed as a toxinosis. Mt Sinai J Med 44: 33–41. 321946

[26] Fasano A, Noriega FR, Liao FM, Wang W, Levine MM (1997) Effect of shigella enterotoxin 1 (ShET1) on rabbit intestine in vitro and in vivo. Gut 40: 505–511. 9176079 10.1136/gut.40.4.505 PMC1027126

[27] Kotloff KL, Pasetti MF, Barry EM, Nataro JP, Wasserman SS, et al (2004) Deletion in the Shigella enterotoxin genes further attenuates Shigella flexneri 2a bearing guanine auxotrophy in a phase 1 trial of CVD 1204 and CVD 1208. J Infect Dis 190: 1745–1754. 15499528 10.1086/424680

[28] Bennish ML, Salam MA, Wahed MA (1993) Enteric protein loss during shigellosis. Am J Gastroenterol 88: 53–57. 8420274

[29] Constantinescu AR, Bitzan M, Weiss LS, Christen E, Kaplan BS, et al (2004) Non-enteropathic hemolytic uremic syndrome: causes and short-term course. Am J Kidney Dis 43: 976–982. 15168377 10.1053/j.ajkd.2004.02.010

[30] Bennish ML KI, Khan AM, Azad AK, Robertson G (1988) Hyponatremia in shigellosis: incidence and mechanisms. 28th Interscience Conference on Antimicrobial Agents and Chemotherapy (ICAAC). Los Angeles, California.

[31] Legros D, Paquet C, Dorlencourt F, Saoult E (1999) Risk factors for death in hospitalized dysentery patients in Rwanda. Trop Med Int Health 4: 428–432. 10444318 10.1046/j.1365-3156.1999.00413.x

[32] Nathoo KJ, Porteous JE, Siziya S, Wellington M, Mason E (1998) Predictors of mortality in children hospitalized with dysentery in Harare, Zimbabwe. Cent Afr J Med 44: 272–276. 10910572

[33] van den Broek JM, Roy SK, Khan WA, Ara G, Chakraborty B, et al (2005) Risk factors for mortality due to shigellosis: a case-control study among severely-malnourished children in Bangladesh. J Health Popul Nutr 23: 259–265. 16262023

[34] Mitra AK, Engleberg NC, Glass RI, Chowdhury MK (1990) Fatal dysentery in rural Bangladesh. J Diarrhoeal Dis Res 8: 12–17. 2229985

[35] Kavaliotis J, Karyda S, Konstantoula T, Kansouzidou A, Tsagaropoulou H (2000) Shigellosis of childhood in northern Greece: epidemiological, clinical and laboratory data of hospitalized patients during the period 1971–96. Scand J Infect Dis 32: 207–211. 10826910 10.1080/003655400750045358

[36] Bardhan P, Faruque AS, Naheed A, Sack DA (2010) Decrease in shigellosis-related deaths without Shigella spp.-specific interventions, Asia. Emerg Infect Dis 16: 1718–1723. 21029529 10.3201/eid1611.090934 PMC3294502

[37] Bennish ML, Khan WA, Begum M, Bridges EA, Ahmed S, et al (2006) Low risk of hemolytic uremic syndrome after early effective antimicrobial therapy for Shigella dysenteriae type 1 infection in Bangladesh. Clin Infect Dis 42: 356–362. 16392080 10.1086/499236

[38] Huang IF, Chiu CH, Wang MH, Wu CY, Hsieh KS, et al (2005) Outbreak of dysentery associated with ceftriaxone-resistant Shigella sonnei: First report of plasmid-mediated CMY-2-type AmpC beta-lactamase resistance in S. sonnei. J Clin Microbiol 43: 2608–2612. 15956372 10.1128/JCM.43.6.2608-2612.2005 PMC1151904

[39] National Institute of Population Research and Training (NIPORT), Mitra and Associates a, International I (2013) Bangladesh Demographic and Health Survey 2011. Dhaka, Bangladesh and Calverton, Maryland, USA: NIPORT, Mitra and Associates, and ICF International.

[40] Trofa AF, Ueno-Olsen H, Oiwa R, Yoshikawa M (1999) Dr. Kiyoshi Shiga: discoverer of the dysentery bacillus. Clin Infect Dis 29: 1303–1306. 10524979 10.1086/313437

[41] Barrett-Connor E, Connor JD (1970) Extraintestinal manifestations of shigellosis. Am J Gastroenterol 53: 234–245. 5435635

[42] Chisti MJ, Faruque AS, Khan WA, Das SK, Zabed MB, et al (2010) Characteristics of children with Shigella encephalopathy: experience from a large urban diarrhea treatment center in Bangladesh. Pediatr Infect Dis J 29: 444–447. 20016394 10.1097/INF.0b013e3181cb4608

[43] Biedner B, Rosenblatt I, Dugan R, Yassur Y (1987) Corneal ulcer caused by Shigella flexneri in an infant. Am J Ophthalmol 104: 90. 10.1016/0002-9394(87)90304-7 3300355

[44] Baiulescu M, Hannon PR, Marcinak JF, Janda WM, Schreckenberger PC (2002) Chronic vulvovaginitis caused by antibiotic-resistant Shigella flexneri in a prepubertal child. Pediatr Infect Dis J 21: 170–172. 11840089 10.1097/00006454-200202000-00019

[45] Papasian CJ, Enna-Kifer S, Garrison B (1995) Symptomatic Shigella sonnei urinary tract infection. J Clin Microbiol 33: 2222–2223. 7559987 10.1128/jcm.33.8.2222-2223.1995 PMC228374

[46] Mazumder RN, Salam MA, Ali M, Bhattacharya MK (1997) Reactive arthritis associated with Shigella dysenteriae type 1 infection. J Diarrhoeal Dis Res 15: 21–24. 9308297

